# Impact of antibiotics, iron oxide, and sodium sulfate on microbial community composition in laboratory-built municipal solid waste microcosms

**DOI:** 10.1371/journal.pone.0318351

**Published:** 2025-01-28

**Authors:** Judy Malas, Sarah C. Khoury, Michael Tanzillo, Gracie A. Fischer, Jean E. Bogner, D’Arcy R. Meyer-Dombard

**Affiliations:** Department of Earth and Environmental Sciences, University of Illinois at Chicago, Chicago, IL, United States of America; Tsinghua University, CHINA

## Abstract

Municipal solid waste (MSW) landfills represent underexplored microbial ecosystems. Landfills contain variable amounts of antibiotic and construction and demolition (C&D) wastes, which have the potential to alter microbial metabolism due to biocidal or redox active components, and these effects are largely underexplored. To circumvent the challenge of MSW heterogeneity, we conducted a 65-day time series study on simulated MSW microcosms to assess microbiome changes using 16S rRNA sequencing in response to 1) Fe(OH)_3_ and 2) Na_2_SO_4_ to represent redox active components of C&D waste as well as 3) antibiotics. The addition of Fe(OH)_3_ altered the overall community composition and increased Shannon diversity and Chao1 richness. The addition of a mixture of seven antibiotics (1000 ng/L each) altered the community composition without affecting diversity metrics. Sulfate addition had little effect on microbial community composition or diversity. These results suggest that the microbial community composition in fresh MSW may be significantly impacted by influxes of iron waste and a single application of antibiotics.

## 1. Introduction

Microbial communities within landfills mediate the decomposition of Municipal Solid Waste (MSW) and play an important role in global elemental cycles [1]. Landfills are significant contributors to the global carbon cycle and are an important source of anthropogenic methane emissions [2–4]. Conditions in landfills range in pH from 4.5 to neutral, temperatures from ambient up to 120°C, and depths from 15 m to 100 m [5, 6]. Fresh waste may be nutrient-dense due to food waste and oxygen availability, but buried waste is quickly depleted in oxygen and cut off from photosynthetic primary productivity. Due to the diversity of potential metabolic niches and environmental gradients, landfills also serve as valuable resources for biotechnology [7–9].

Studying the microbial communities within landfills presents a unique challenge due to the heterogeneity of landfill ecosystems. Previous studies have attempted to characterize the environmental drivers of microbial communities within landfills using internal liquids (leachate), gas, buried refuse, or intercalated soils [10–14]. While these studies are useful for comparing microbial communities across different climatic locations, ages, and depths, they can provide only a snapshot of specific locations within a given landfill at a given time. Landfills vary with regard to management strategies, inputs, moisture content, available nutrients, cover soil, and their local climate [11, 12, 15, 16]–all of which can impact the microbial community composition and thus present challenges for experimental reproducibility. Several studies have used homogenized landfill waste or fresh refuse in laboratory-based reactors to circumvent issues of heterogeneity [17–20]. However, information about waste input and waste age is lost in most cases.

Recognizing the challenges associated with studying drivers of microbial community composition and diversity in MSW landfills, we undertook a simplified model system to investigate the microbial dynamics of the MSW ecosystem and potential sources of disruption to typical microbial metabolic processes in MSW. Barlaz, Milke, and Ham (1989) observed four degradation stages under ideal conditions in laboratory bioreactors [18]: 1) the aerobic phase, wherein oxygen and nitrate were depleted, and sugars were converted to CO_2_ and water; 2) the anaerobic acid phase characterized by an accumulation of carboxylic acids and a decrease in pH; 3) the accelerated methane production phase accompanied by an increase in pH; and 4) the decelerated methane production phase. Improved understanding of the waste degradation process can facilitate effective landfill management, enhance prediction of greenhouse gas emissions, and support the implementation of environmentally sustainable practices such as biogas capture [21]. Here, we investigated the potential for different waste streams to alter the bacterial population expected during terminal phases of waste degradation and thus change the progression of waste degradation.

Many U.S. landfills accept large quantities of construction and demolition (C&D) waste as recycling rates are highly variable and, importantly, periodic disaster debris (i.e., from hurricanes) must be cleared quickly. In 2018, approximately 600 million tons of C&D debris were generated, 24% (144 million tons) of which were sent to U.S. landfills, including gypsum drywall (13.2 million tons) and total metals (1.1 million tons) [22]. While some landfill sites in the United States are designated industrial C&D landfills, MSW landfills can also accept C&D waste if it is non-hazardous. Both iron and sulfate are redox-active species present in C&D waste that can be used for various microbial metabolisms [1].

The addition of Fe^2+^ or Fe^3+^ has been shown to increase methane oxidation in digested sewage [23], as methane oxidation can be coupled to the reduction of Fe^3+^ [24]. In anoxic rice paddy soils, the addition of ferrihydrite (Fe^3+^) has been shown to suppress methanogenesis [e.g., 25]. Similarly, in anaerobic, wet soil environments, sulfate reduction may be coupled to organic carbon degradation, anaerobic methane oxidation, and hydrogen consumption–and therefore, sulfate reduction may compete with methanogenesis [26]. In addition to C&D waste, sulfate within landfills can come from *in situ* geology/soils at the site of landfill development (i.e., subaerial oxidation at former sulfide ore mine pit; marine-derived sulfate-rich coastal sediments) or sulfate-rich natural soils transported to site for use as cover materials. Historically, some landfill sites have used "fines" from recycled construction debris with relatively high concentrations of gypsum as an alternative daily cover soil [27]. This practice rapidly produced high levels of H_2_S in the recovered biogas, which also reduced relatively quickly due to the depletion of the fine-grained sulfate. Previous studies have differed with respect to the quantification of sulfate effects on methanogenesis or anaerobic methane oxidation in landfills [23, 28, 29], and few studies have investigated the influence of redox-active components of C&D waste on the MSW microbiome with modern sequencing technologies.

Landfills in the United States also contain at least 101 different prescription and non-prescription pharmaceuticals, industrial chemicals, household chemicals, steroid hormones, and animal and plant sterols [30]. Antibiotic consumption is increasing worldwide, and landfills are one of the primary routes for the disposal of these drugs [31]. Landfills have been found to be a major reservoir for antibiotics and antibiotic resistance genes (ARGs) [31–33]. Both antibiotics and ARGs have been found in high concentrations in environments surrounding landfills due to leaky liners or discharge of treated leachate effluent [31, 34–36]. The concentration of antibiotics within landfill leachates varies widely, from 0 ng/L up to over 250,000 ng/L, and 1000 ng/L is a commonly observed concentration in landfill leachates [31, 37]. Antibiotics present in the environment have been shown to alter microbial community composition, decrease microbial diversity, and contribute to the development of ARGs; however, the impact of antibiotics in different environments depends on several factors, including the type of antibiotic as well as the pH, organic carbon content, type of soil, and the resident microbial community [38–40]. Several studies have quantified the concentrations of antibiotics and ARGs in landfills [30, 41–44], and one report found that the addition of sulfamethazine to landfill refuse increased ARG levels tenfold after a 65-day incubation while reducing the functional capacity for denitrification [45]. Apart from the work by Wu et al. (2017), there is a relative dearth of experimental works investigating the influence of antibiotics on the MSW microbiome.

Though research on the microbial dynamics of landfill systems has increased significantly in the last 5 years, more experimental work is needed to determine the influence of bioactive wastes on the MSW microbial community. Here, microcosms were constructed for laboratory incubations of fresh refuse from predetermined amounts of raw material (e.g., paper, plastic, food waste) and incubated for 65 days at 45°C. We used this model system to test the redox active components of construction and demolition (C&D) waste and antibiotics. The treatments, each introduced separately, were 1) Fe(OH)_3_, 2) Na_2_SO_4_, and 3) antibiotics. Changes to the microbial community were monitored via 16S rRNA sequencing before and after adding the three "contaminant" treatments. These inputs were selected as they could change microbial metabolism by offering alternative energy sources in the case of Fe(OH)_3_ or Na_2_SO_4_ or eradicating groups of organisms in the case of antibiotics. The Fe(OH)_3_ was added as an insoluble substrate to resemble the addition of solid metal construction waste that has rusted after landfilling. The sulfate addition was intended to mimic the addition of gypsum wallboard waste.

## 2. Materials and methods

### 2.1. Lab-built MSW microcosms

MSW microcosms were constructed in 100 mL wide-mouth, flat-bottom glass media bottles. Screw caps included a silicon septum to allow the release of produced gas. An artificial MSW formulation was developed that was made up of small pieces of waste components: 2 g shredded paper (length 2 cm ± 1, width 0.75 cm ± 0.3), 0.8 g aluminum (length 1.7 cm ± 0.6, width 1.5 cm ± 0.4), 2 g plastic (length 2.2 cm ±1, width 1 cm ± 0.5), and 2.5 g dry leaves (length 2.5 cm ± 1, width 1.5 ± 0.6). As well as 10.8 g food waste (fruit, vegetables, grains, peanut butter), 2 g sawdust, 2.4 g soil, and 4 g sand. The total weight of solid waste inputs was 26.5 g. These components were based roughly on Environmental Protection Agency statistics for waste generation [46]; however, the relative proportion of paper waste had to be decreased due to volumetric constraints. The food waste was homogenized in a commercial blender to maintain consistency in individual microcosms, as previous work has suggested that food waste may be the primary contributor to the microbial composition in fresh refuse [47]. The non-food inputs were not finely ground.

**Fig 1:**
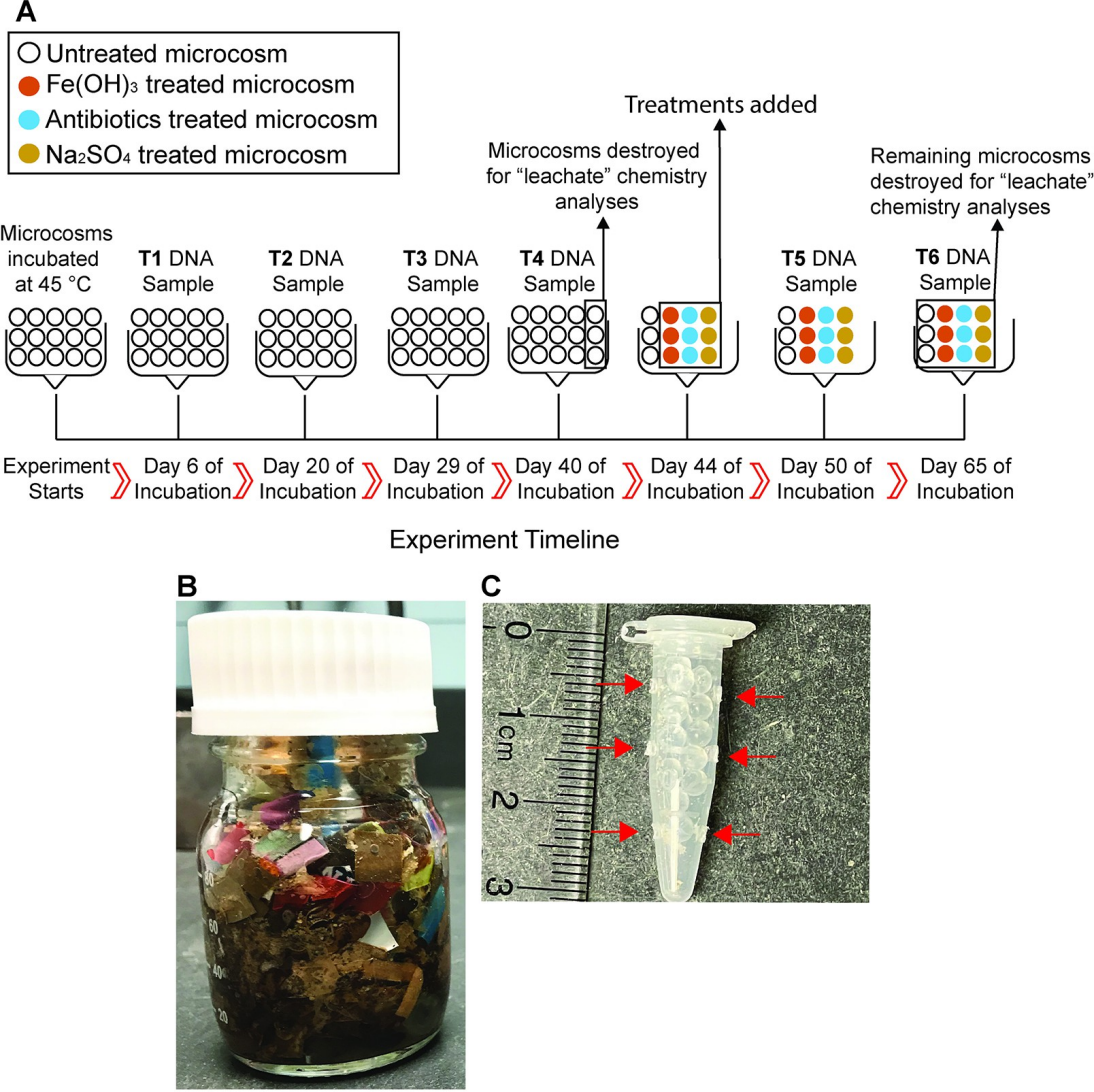
A) Experimental setup and timeline. Each circle represents one microcosm. Microcosm bottles are destroyed during simulated leachate chemistry analyses. T1, T2, T3, T4, T5, and T6 correspond to time points when DNA was sampled during the experiment. B) Depiction of assembled microcosm in 100 mL bottle. C) Centrifuge tube with glass beads, which are inserted into the microcosm during assembly. Arrows point to perforations in the tubes through which microcosm liquid can flow to the beads.

In order to collect samples for downstream DNA extraction in a reproducible manner, four perforated, 0.6 mL centrifuge tubes filled with sterile 3 mm spherical Soda-lime glass beads (~75% SiO_2_; Fisher Scientific catalog # 11-312A) were inserted into the microcosms at the time of construction and embedded fully in the solid waste (Fig 1). The tubes were distributed randomly within the solid waste. The microorganisms on the beads represent those transferred through the microcosm in the fluid that then colonized the glass beads. Glass beads were used because they are a non-reactive surface chemically abundant in soils (SiO_2_), typically an average of 72 weight percent of soil, that would not directly impact the metabolic choices of the microorganisms [48]. Each 0.6 mL centrifuge tube fits 16 glass beads, for a total of 64 glass beads per microcosm. In addition, 70 mL of sterile water was added to the bottles to cover the waste contents (reaching the 80 mL fill line on the bottles). The water ensured that the organisms were not transport-limited within the waste, provided a solvent for even distribution of contaminant treatments, and served as simulated leachate for spectrophotometric detection of dissolved ions. Glass bottles, deionized water, glass beads, and 0.6 mL tubes were autoclaved at 121°C for 55 min prior to assembling the microcosms.

Microcosms were incubated in water baths at 45°C to keep temperatures constant for 65 days to capture the first three phases of waste degradation previously observed in laboratory-based reactors under similar incubation temperatures [18]. A temperature of 45°C was selected as it is in the typical operating range for landfills, though specific temperatures within landfills are dependent on local climate [49].

### 2.2. Contaminant treatments

Microcosms were set up in triplicate, with one set of triplicate bottles for each experimental treatment, as well as six control group bottles, for a total of 15 microcosms (Fig 1). The microcosms were maintained untreated for 44 days. This incubation time reflects the approximate onset of the production of methane [18], the terminal product of waste degradation. The addition of contaminants at this phase was intended to determine whether bioactive contaminants alter the progression of waste degradation. After 44 days, three bottles each were amended with the following treatments: 0.5 g Fe(OH)_3_, 0.21 g Na_2_SO_4_ (~2000 mg/L sulfate), or a mixture of each of seven antibiotics to a final concentration of 1000 ng/L total per antibiotic. The antibiotics used were Amoxicillin, Cephalexin hydrate, Amoxicillin trihydrate: Potassium Clavalante (4:1 penicillin combination), Azithromycin, Sulfamethoxazole, Bacitracin, and Tetracycline. All antibiotics were purchased from Sigma-Aldrich. Solutions of antibiotics were made with autoclaved DI water, and the solutions were filter sterilized with a 0.2 micron Whatman Puradisc syringe filter before incorporation into the microcosms. The Fe(OH)_3_ and a solution of Na_2_SO_4_ were autoclaved at 121°C for 55 min prior to addition. The concentrations selected for antibiotic and sulfate additions were based on previous findings in landfill leachate [12, 41].

### 2.3. Microcosm simulated leachate chemistry

After 40 days (T4), three control microcosms were destructively sampled to analyze sulfide, sulfate, total iron, and ferrous iron from unfiltered microcosm liquids (simulated leachate). The remaining three control and nine treated bottles were sampled for chemistry at the end of the 65-day period (T6). Sulfide, sulfate, total iron, ferrous iron (Fe^2+^), and pH were measured in all microcosms at T6 after 65 days of incubation. Paper strips were used for pH measurements to preserve simulated leachate for remaining chemical analyses. Data were obtained using a portable field HACH spectrophotometer DR2700, as described previously [50].

### 2.4. DNA sampling, extraction, and sequencing

DNA was sampled at six timesteps after 6 (T1), 20 (T2), 29 (T3), 40 (T4), 50 (T5), and 65 (T6) days (Fig 1). A DNA sample was not collected at the start of the incubation as the beads were sterilized prior to microcosm assembly. Sterilized forceps were used to extract a perforated tube from the microcosm, and six glass beads were collected from the microcosm per sample and deposited directly into a PowerBead tube from the QIAGEN DNeasy PowerMax Soil Kit, which was used to extract the DNA according to the manufacturer’s instructions. Additional sterilized deionized water was added to the 80 mL fill line to keep the waste submerged if water was lost during sampling. The DNA sampling was conducted in an anaerobic chamber so as not to introduce oxygen after initial microcosm construction to mimic the conditions of buried waste. PowerBead tubes with glass beads were stored at -20°C until DNA extraction.

Genomic DNA was PCR amplified with primers CS1_515 (GTGCCAGCMGCCGCGGTAA) and CS2_806R (GGACTACHVGGGTWTCTAAT) (Caporaso et al. 2011); region-specific primers used to amplify the V4 region of the 16S rRNA gene. The primers contained 5’ common sequence tags known as common sequence 1 (CS1) and 2 (CS2), as described previously [51]. Amplicons were generated using a two-stage PCR amplification protocol [52]. During the second PCR amplification, each sample received a unique 10-base barcode obtained from the Access Array Barcode Library for Illumina (Fluidigm, South San Francisco, CA; Item# 100–4876). These AccessArray primers contained the CS1 and CS2 linkers at the 3’ ends of the oligonucleotides. Libraries were then pooled in equal volumes, and the pool was purified using an AMPure XP cleanup protocol (0.6X, vol/vol; Agencourt, Beckmann-Coulter) to remove fragments smaller than 300 bp. The pooled libraries, with a 15% PhiX sequencing control spike-in, were loaded onto an Illumina MiniSeq mid-output flow cell (2x153 paired-end reads). Based on the distribution of reads per barcode, the amplicons (before purification) were re-pooled to generate a more balanced distribution of reads. The re-pooled libraries, with a 15% PhiX spike-in, were loaded onto a Miniseq flow cell and sequenced (2x153 paired-end reads). Fluidigm sequencing primers targeting the CS1 and CS2 linker regions were used to initiate sequencing. De-multiplexing of reads was performed on the instrument. Library preparation, pooling, and sequencing were performed at the Genome Research Core within the Research Resources Center at the University of Illinois at Chicago. Raw data files were deposited to the Sequence Read Archive (SRA) and are available under BioProject accession number PRJNA950411.

### 2.5. Data analyses

Paired-end reads were merged with PEAR (v.0.9.11; [53]). After merging, the reads were quality processed to remove sequences with low-quality scores and binned into amplicon sequence variants (ASVs) using DADA2 (v.1.22.0; [54]), according to the pipeline outlined by Callahan et al. (2016b) with minor adjustments to account for differences in read length [55]. Quality-filtered reads were used to construct an amplicon sequence variant (ASV) table using the SILVA database v.138. Taxonomic composition analyses were performed using *Phyloseq* (v.1.38.0; [56]) and visualized using ggplot2 (v3.4.4; [57]) in R (v 4.1.3).

Beta diversity of the samples was calculated using both the Bray-Curtis and Euclidean distance metrics. As rarefying sequence data (cutting all sample libraries to the same read number) has been advised against [58], the Bray-Curtis distance metric is a popular metric recommended for untransformed data [59]. However, this method does not account for potential bias due to differences in library sizes. Therefore, as an alternative to rarefaction, Euclidean distances were also calculated after applying a variance stabilizing transformation to account for the differences in number of reads per sample in DESeq2 (v 1.34.0) using code published by Lee et al. (2019) [58, 60, 61]. A Non-metric Multidimensional Scaling (NMDS) plot or a Principal coordinates analysis (PCoA) plot was made for the Bray-Curtis and Euclidean distances, respectively. To assess statistical significance, a permutational multivariate analysis of variance (PERMANOVA) test was conducted using both distance metrics within the Vegan package (v2.6–4) in R [62]. PERMANOVA tests were calculated based on 10,000 permutations and p values were adjusted for multiple testing using the Bonferroni method. A multivariate homogeneity of group dispersions (PERMDISP2) test was conducted in conjunction with each PERMANOVA to ensure that the variance within groups did not exceed the variance between groups.

Alpha diversity metrics were calculated within the Microbiome package (v1.16.0;[63]). A Wilcoxon rank-sum test was used to assess the statistical significance of alpha diversity between groups. DA testing was conducted with Analysis of Compositional Microbiomes with Bias Correction (ANCOM-BC) (v.2.0.3; [64]). ASVs were aggregated at the Genus level and P values were adjusted for multiple testing using the Holm method for the ANCOM-BC analyses.

## 3. Results

### 3.1. Simulated leachate chemistry

Sulfide, sulfate, total iron, ferrous iron (Fe^2+^), and pH were measured in the untreated microcosms after 40 days of incubation. Subsequently, these measurements were conducted in all microcosms after 65 days of incubation (Table 1). The pH of all microcosms, regardless of treatment, was found to be ~5.5 at 40 days as well as at the end of the incubation period. Sulfate was only detected in the sulfate-treated microcosms, likely sourced from the added treatment, with an average of 2000 mg/L. This concentration was roughly equivalent to the ~2000 mg/L sulfate added to each microcosm. In contrast, dissolved total iron in the Fe(OH)_3_ treatments was found to be less than the control microcosms at 65 days, suggesting that the addition of iron triggered precipitation of iron from solution or chelation / complex-formation of the iron.

**Table 1:** Average concentrations of total iron, ferrous iron, sulfide, sulfate, and pH in triplicate live microcosms. Data are reported in mg/L for all chemical species. N.d. = not detected.

	Total Iron	Ferrous Iron	Sulfide	Sulfate	pH
Control 40 days	7.1 ± 2	7.8 ± 2	3.9 ± 1.4	93 ± 144	5.5
Control 65 days	78 ± 31	62 ± 35	4.9 = 2.6	*N*.*d*.	5.5
Fe(OH)_3_ 65 days	22 ± 9	15 ± 8	4.2 ± 2.2	*N*.*d*.	5.5
Antibiotics 65 days	52 ± 11	38 ± 13	7.4 ± 2.5	*N*.*d*.	5.5
Na_2_SO_4_ 65 days	118 ± 65	100 ± 56	6.9 ± 2.1	2000 ± 100	5.5

### 3.2. Microbial community composition

A total of 82 DNA samples passed quality control. Two samples had to be removed either because they did not amplify by PCR prior to sequencing or due to low read counts (< 850 reads per sample). A total of 1564 ASVs were detected. Taxonomic classification with the SILVA database v.138 was possible to the genus level, however, many ASVs were only classified to the phylum or order level.

**Fig 2:**
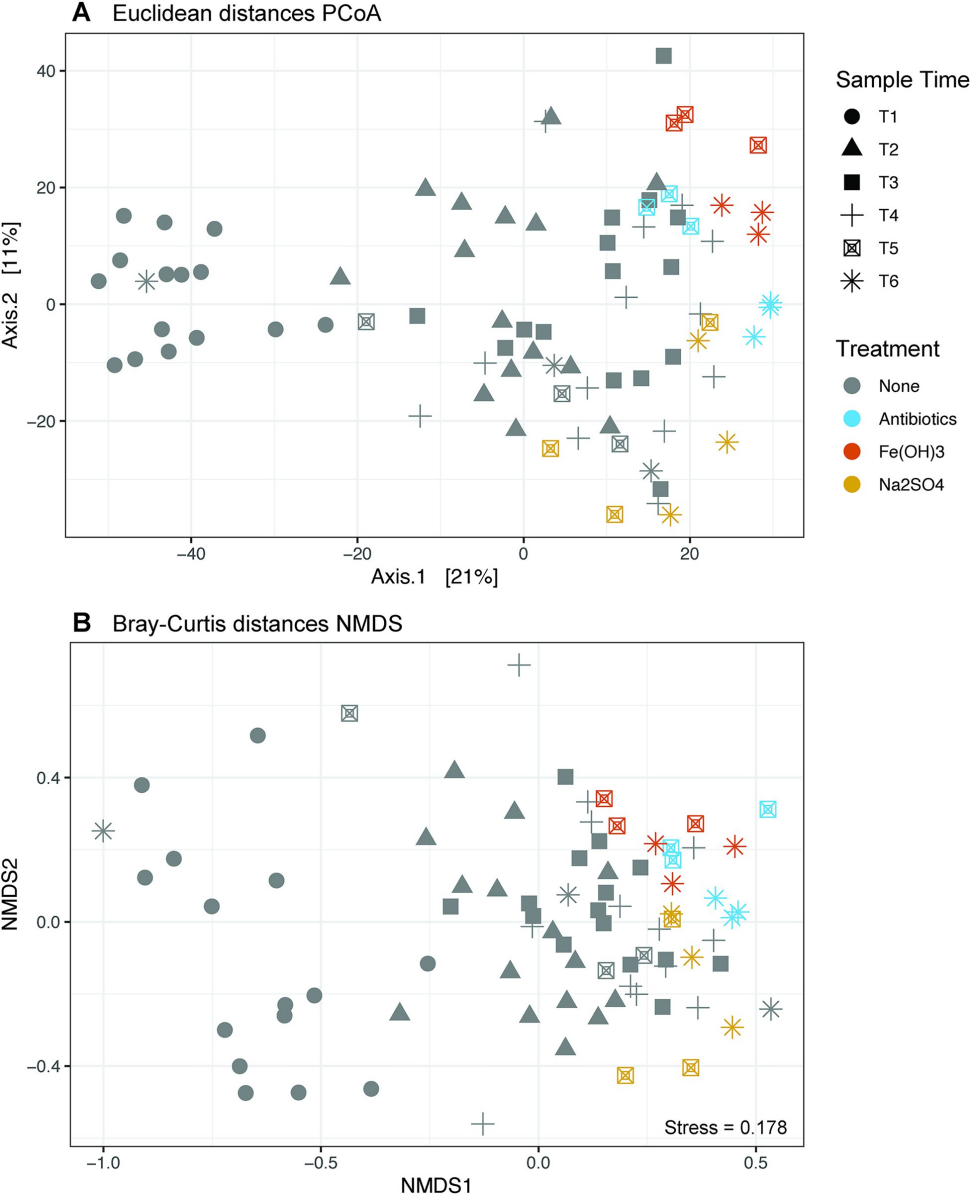
A) PCoA of Euclidean distances. B) NMDS of Bray-Curtis distances.

The microbial community composition of the microcosms at the start of the incubation (T1; day 6 of incubation) is distinct from all other sampling time points (Fig 2). The microbial community composition was largely established by T2, as the composition of the untreated microcosms did not exhibit significant differences during the other sampled time points (Table 2). The NMDS and PCoA plots show T1 samples clustering separately from the rest of the samples (Fig 2). Pairwise PERMANOVA testing on Euclidean distances shows that the difference (beta-diversity) between T1 and other sample time groups is significant (p < 0.05), aside from T6 (Table 2). Though the PERMANOVA indicates T6 is not significantly different from T1, this result may be due to the untreated control outlier sample in T6, which can be seen in the NMDS/PCoA plots (Fig 2).

**Table 2:** Results of pairwise PERMANOVA tests for Euclidean distances between groups. P values were adjusted for multiple testing using the Bonferroni method. Pairwise comparisons between sample times (i.e. T1-T6) include only untreated microcosms. Post-treatment groups include both T5 and T6.

Untreated controls	Df	SumsOfSqs	F.Model	R^2^	p.adjusted	sig
T1 vs T2		1	14621.09	6.89	0.2	0.0015	**
T1 vs T3	1	22020.01	10.95	0.28	0.0015	**
T1 vs T4	1	22030.91	10.3	0.28	0.0015	**
T1 vs T5	1	7003.33	3.45	0.18	0.0210	*
T1 vs T6	1	5328.73	2.52	0.32	0.0855	
T2 vs T3	1	2080.93	0.97	0.14	1.0000	
T2 vs T4	1	4203.57	1.83	0.07	0.1995	
T2 vs T5	1	3123.15	1.37	0.08	1.0000	
T2 vs T6	1	3581.48	1.51	0.09	0.7919	
T3 vs T4	1	1834.05	0.84	0.03	1.0000	
T3 vs T5	1	2742.97	1.32	0.08	1.0000	
T3 vs T6	1	3709.61	1.71	0.10	0.3149	
T4 vs T5	1	1990.06	0.85	0.06	1.0000	
T4 vs T6	1	3022.76	1.25	0.08	1.0000	
T5 vs T6	1	1351.63	0.50	0.11	1.0000	
Post-treatment
Antibiotics vs None	1	7046.1	3.51	0.26	0.0138	*
Fe(OH)_3_ vs None	1	9015.45	4.52	0.31	0.0150	*
Na_2_SO_4_ vs None	1	3942.46	1.93	0.16	0.0792	

** P < 0.01

* P < 0.05

**Fig 3:**
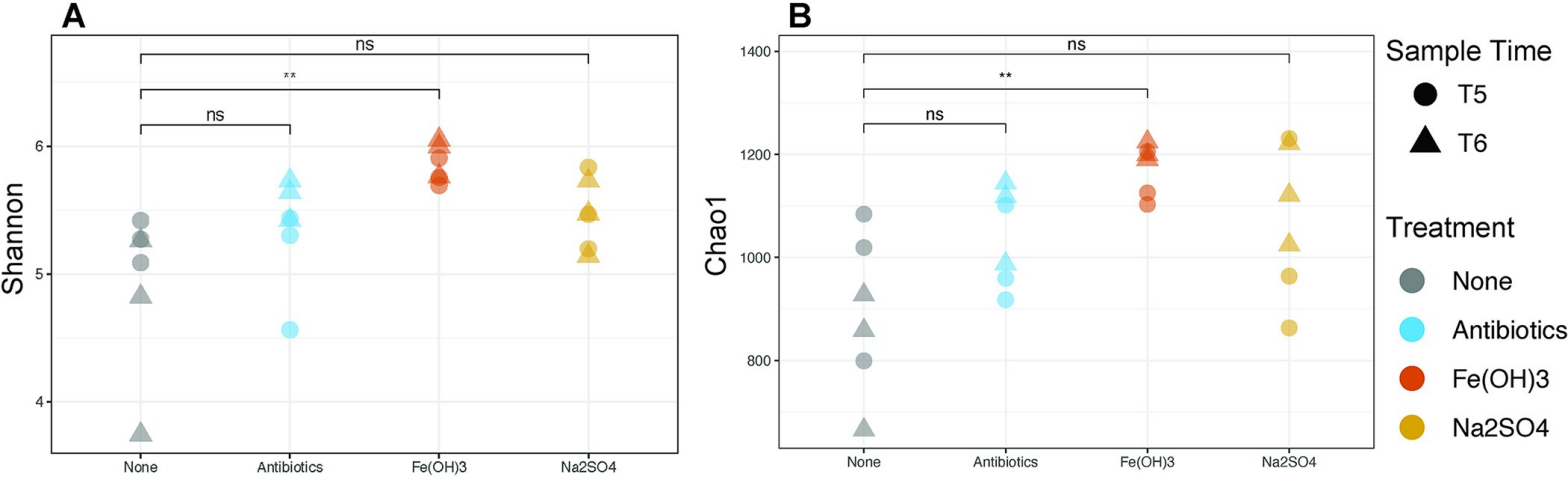
A) Chao1 B) Shannon-Weaver alpha diversity metrics. Wilcoxon-rank sum testing was conducted to test for significant differences in diversity between the untreated control and each treatment. P <0.01 is indicated by "**", not significant is indicated "ns".

Contaminant treatments produced a shift in the microbial community, particularly in the Fe(OH)_3_ and antibiotic treatments compared to the untreated control. The Fe(OH)_3_ treatment had the greatest influence on the microbial community, reflected in the highest R^2^ of the three treatment groups relative to the untreated microcosms (R^2^ = 0.31; Table 2) and a significant increase in Shannon and Chao1 indexes (Fig 3). The antibiotic treatment significantly influenced the microbial community composition to a lesser degree than the iron treatment (Fig 2; Table 2). Shannon and Chao1 alpha diversity metrics of the untreated control and the antibiotic group were not significantly different (Fig 3). This indicates that while some taxa may have been substituted due to antibiotic treatment, the overall alpha diversity within the microcosms did not increase or decrease significantly due to treatment. The Na_2_SO_4_ treatment did not significantly impact the community or diversity (Table 2; Fig 3). The statistical significance of treatment on community composition were consistent when using either the Bray-Curtis or Euclidean distance metric (Tables 2 and 3).

**Table 3:** Results of pairwise PERMANOVA results for Bray-Curtis distances between groups after treatment addition. Results include both T5 and T6. P values were adjusted for multiple testing using the Bonferroni method.

Post-Treatment	Df	SumsOfSqs	F.Model	R^2^	p.adjusted	sig
None vs Antibiotics	1	0.99	5.05	0.07	0.0030	**
None vs Fe(OH)_3_	1	0.79	4.1	0.06	0.0072	**
None vs Na_2_SO_4_	1	0.51	2.65	0.04	0.0804	

** P < 0.01

* P < 0.05

### 3.3. Important taxa

**Fig 4:**
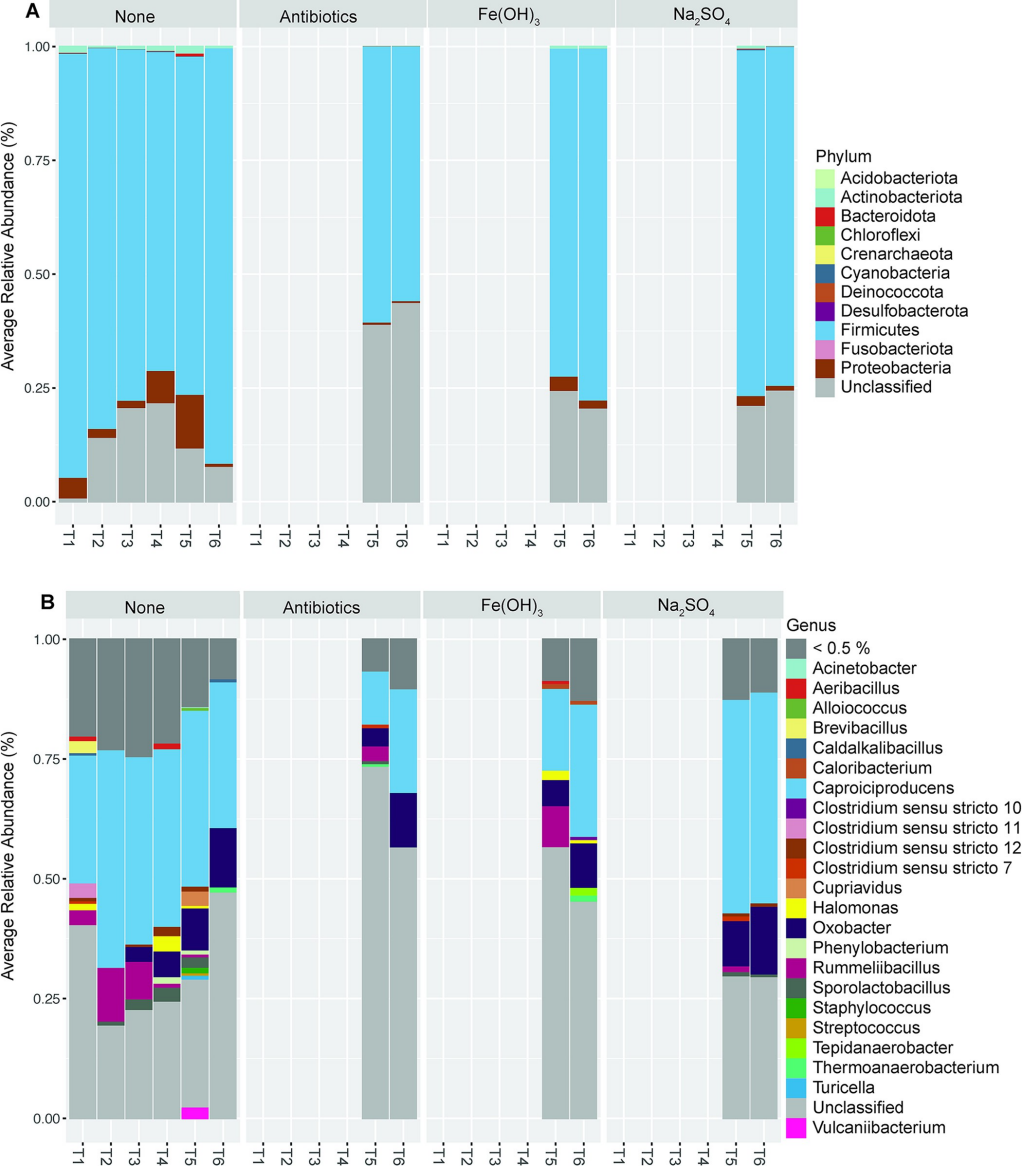
Average relative abundance of taxa. A) shows Phylum level classification and B) shows Genus level classification. Each stacked bar represents the average relative abundance for microcosms replicates at each sampling time. Treatments were added at after T4.

As shown in Fig 4A, Bacteria dominated the sequenced taxa in all microcosms, with the archaeal phylum Crenarchaeota detected in negligibly small amounts (mean of 0.001%). Taxa of the phylum Firmicutes were most abundant in all microcosms, followed by Proteobacteria and Actinobacteriota with a mean of 79%, 2.98%, and 0.78%, respectively across all microcosms (Fig 4A). The genus *Caproiciproducens* within the phylum Firmicutes was a dominant taxon of nearly all the microcosms from the beginning (T1, 6 days) to the end (T6, 65 days) of the incubation period (Fig 4B). There was a higher proportion of low abundance (<0.5%) genera in the microcosms at T5 and T6 than at the start of the incubation at T1 and T2 (Fig 4B), suggesting that lower abundance taxa were driving the compositional differences between treatment groups. This may have been the case in the Fe(OH)_3_ treatment, as the Chao1 richness, which is sensitive to the presence of rare species, was significantly higher in these treatments than in the untreated control (Fig 3B).

**Fig 5:**
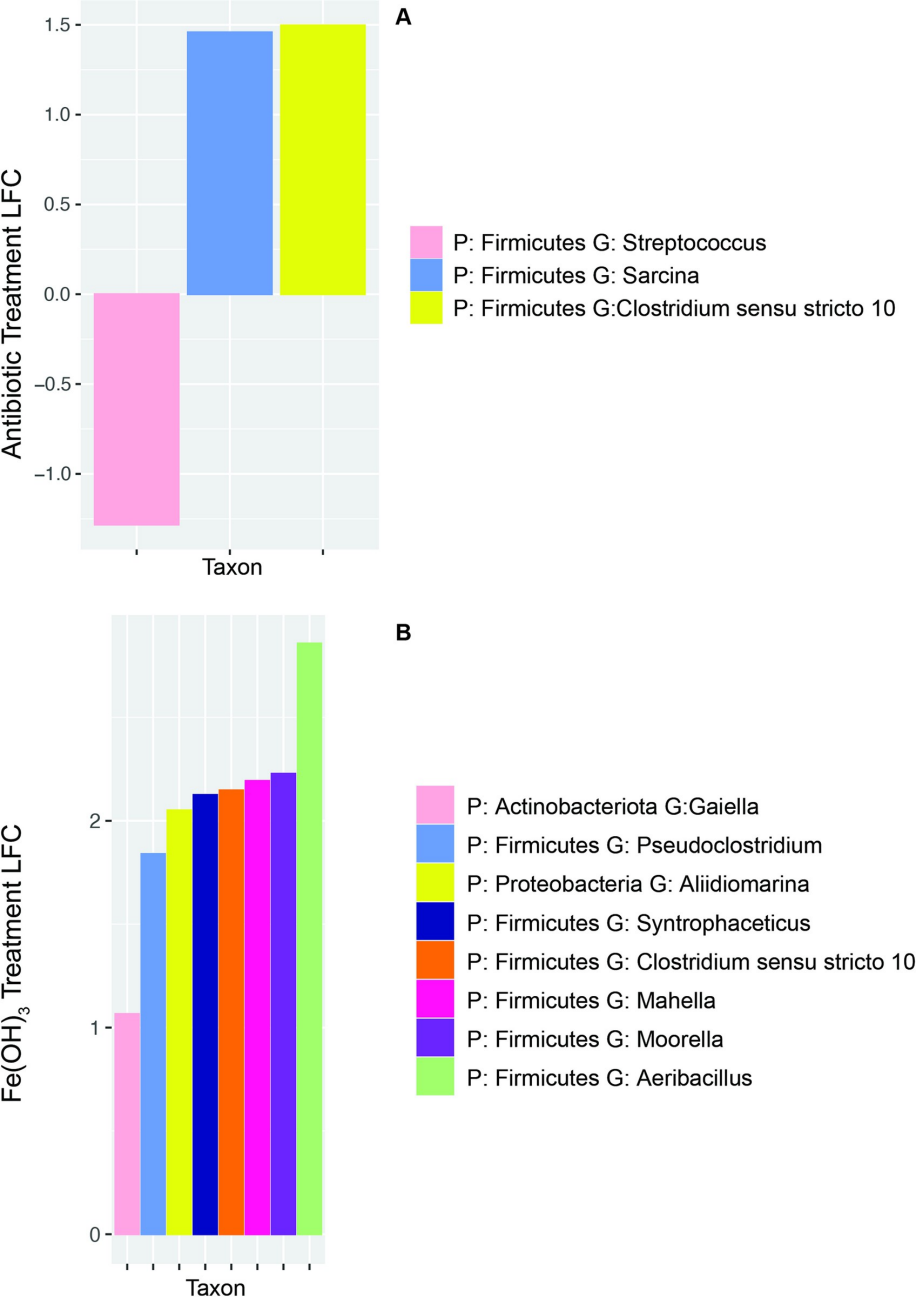
Differentially abundant taxa as determined by ANCOM-BC testing for a) antibiotic treatment and B) Fe(OH)_3_ treatment compared to untreated control microcosm. Natural log fold change reported on the Y axes. P = Phylum, G = Genus.

Differential abundance testing was conducted to determine which taxa increased or decreased after the treatments. An ANCOM-BC test was conducted on the treated microcosms compared to the untreated control microcosms, both groups pooled at T5 and T6. Taxa were aggregated at the genus level, and a total of 103 genera were tested for differential abundance. Taxa that changed by natural log fold change of greater than | 1 | were considered differentially abundant, and any aggregated taxa that were not classified at the genus level were filtered from the results. Consistent with the PERMANOVA results, the Fe(OH)_3_ treatment resulted in the largest number of differentially abundant taxa post-treatment compared to the untreated control, followed by the antibiotic treatment (Fig 5). The genus *Aeribacillus* displayed the greatest log fold change of 2.86 after iron addition, corresponding to approximately an order of magnitude increase in abundance (ln(2.86) = 1.05). The sulfate treatment did not result in significantly differentially expressed taxa according to the ANCOM-BC test.

Differentially abundant taxa that were classified at the Genus level were further investigated by performing a Wilcoxon test on the log10 abundance in the different treatment groups to validate the ANCOM results. Wilcox testing on the log 10 abundance confirmed that taxa of the following genera were differentially abundant (p < 0.05). Taxa of the genera *Gaiella*, *Pseudoclostridium*, *Aliidiomarina*, *Syntrophaceticus*, *Clostridium sensu stricto 10*, *Moorella*, and *Aeribacillus* were significantly differentially abundant in the iron-treated microcosms (Fig 5B). Taxa of the genera *Streptococcus* and *Sarcina* were significantly differentially abundant in the antibiotic-treated microcosms (Fig 5A), and the genus *Mahella* and *Clostridium sensu stricto 10* was significant in both treatment groups.

## 4. Discussion

Previous work has found that sampling site, sampling depth, landfill age, and geographic context/local climate all influence the microbial community structure within landfills [12, 65–67], which makes assessing the influence of a single variable particularly challenging. Here, we have attempted to circumvent this challenge by utilizing lab-built microcosms simulating fresh MSW. The advantage of this simulated MSW degradation study with lab-built microcosms is that waste inputs can be controlled rather than inferred and DNA can be collected in a reproducible manner across time and across samples. In this study, we have used microcosms that simulated fresh MSW degradation to study the response of the solid-particle-associated microbial community to selected antibiotics and redox-active components of C&D waste.

We observed a higher degree of heterogeneity in the lab-built microcosms at the start of the incubation (T1), however, the community began to stabilize and converge after just 15 days of incubation at T2 (Fig 2; Table 2). Across microcosms, we identified several taxa that are commonly observed in MSW. The bacterial taxa belonging to the phyla Firmicutes, Proteobacteria, Acidobacteriota (Acidobacteria), Actinobacteriota, Fusobacteriota, and Bacteroidota are consistently found in municipal solid waste landfills (reviewed in, [68]; Fig 4A). Bacteria within these phyla are known to be responsible for the hydrolytic and fermentative processes of waste degradation [68]. At the genus level, several higher abundance genera (> 0.5%) identified in the microcosms of this study have previously been identified in landfill systems (Fig 4B), including *Acinetobacter* [13], *Brevibacillus [69], Lactobacillus* [70], *Caproiciproducens* [71], and *Halomonas* [13]. Low abundance genera in the microcosms have also been previously detected in landfills, for example, *Gelria* [66], *Brevundimonas* [72], and *Pseudomonas* [73]. Several other genera in the microcosms have been detected in anaerobic digestors, wastewater, agriculture, and other industrial environments such as the anaerobic, acetogenic taxon *Oxobacter* [74], which was observed in high relative abundance in the live microcosms at the end of the experimental period (Fig 4B).

It is worth noting that while extracting DNA from glass beads within the microcosms enhances the reproducibility of the technique, it may not capture all microorganisms present if they were not transferred via the microcosm fluid. In a microcosm study investigating the interactions between soil minerals and microorganisms in the rhizosphere, it was found that the community composition of quartz minerals was different from the surrounding soil, but that this difference was driven largely by dispersal limitation (individuals cannot move between communities easily), rather than by variable selective pressure (abiotic or biotic pressures selecting for different characteristics) [75]. The authors suggested that the expression of flagella or other motility factors, as well as sufficient soil moisture would support bacterial dispersion [75]. In the current work, the waste components in the microcosms were fully submerged in water which would support the dispersal of microorganisms into/out of the bead tubes, however, some dispersal limitation may still be influencing the resultant microbial community. Dependent on the goals of future research, it may be worthwhile to sample organisms from individual waste components for greater spatial resolution.

### 4.1. Impact of contaminant treatments

Landfills have been identified as a reservoir of antibiotic-resistant genes (ARG), which could pose issues when/if they seep into the surrounding environment [31, 34]. The results of the antibiotic treatment indicated a significant difference in microbial community composition as compared to the untreated control microcosms (Tables 2 and 3). The alpha diversity of the microbial community, as determined by the Shannon diversity and Chao1 metrics, was not significantly decreased compared to the untreated control (Fig 3). These results are concordant with previous work that showed antibiotic exposure did not substantially decrease the diversity but did affect the community structure of the total bacteria in a laboratory incubation of refuse samples collected from an MSW landfill [45]. Wu et al. (2017) found that the addition of sulfamethazine to landfill refuse increased ARG levels by tenfold after 65 days of exposure to antibiotics while reducing the functional capacity for denitrification, however, the concentrations of antibiotics utilized were several-fold higher than the concentration used here at 25 mg/L [45]. Though the presence of ARGs was not directly assessed here, the significant change in microbial community structure despite stable microbial diversity raises potential concern for the development of antibiotic-resistant genes at the dosages used in this study. The long-term impact of repeated antibiotic exposure at various dosages on the microbial community and the development of ARGs should be investigated in future studies. Though landfilling medical waste is generally considered a safer alternative to disposal directly into wastewater via flushing, there are still important safety considerations to consider when landfilling bioactive medical wastes as they can have a potentially negative influence on the surrounding environment and on nearby human populations [31, 76]. Advanced treatment and containment processes to remove and/or prevent the spread of antibiotic waste and ARGs from landfills have been suggested to overcome these challenges [31, 76].

The addition of iron treatment also impacted the microbial community composition as well as increased microbial diversity in comparison to the control microcosms (Table 2; Fig 5).Total soluble iron at the end of the incubation in the Fe(OH)_3_ treated microcosms was found to be less than the control at 65 days, indicating that the addition of oxidized iron stimulates the removal of iron from solution or chelation / complex-formation of the iron (Table 1). This removal of iron after the addition of Fe(OH)_3_ could be a direct result of biomineralization. Alternatively, the Fe^3+^ could have been converted to Fe^2+^ abiotically and subsequently removed from solution by iron-oxidizing bacteria. Indeed, the majority of iron in our landfill bottles was as Fe^2+^ (Table 1), as expected since Fe^2+^ is more soluble and stable at circumneutral pH (pH 5.5–7.2) [77]. All microcosms measured pH 5.5 regardless of treatment or sample time (Table 1). Biological iron redox can be superimposed against abiotic reactions, and oxidation and reduction of iron may occur simultaneously, which makes parsing the contribution of each extremely difficult [78].

Several genera increased significantly after iron addition including *Gaiella*, *Pseudoclostridium*, *Aliidiomarina*, *Syntrophaceticus*, *Clostridium sensu stricto 10*, *Mahella*, *Moorella*, and *Aeribacillus* (Fig 5B). Most of the strains from these genera are capable of fermentation, aside from *Gaiella* and *Aliidiomarina*, which are only capable of respiration [79, 80]. In landfills, acetate is an important intermediate to methanogenesis; acetoclastic methanogens can use acetate directly or, in the syntrophic pathway, acetate is degraded by acetate oxidizing bacteria and the produced hydrogen is then consumed by a methanogen. *Moorella* is an acetogenic and hydrogenogenic bacterium, capable of utilizing insoluble poorly crystalline Fe(III) oxide to oxidize lactate into acetate [81], and therefore potentially contributes important intermediates for methanogenesis. The acetate-oxidizing genus *Syntrophaceticus* also increased after iron addition; species of this genus have been identified as syntrophic partners to methanogens [82]. It has been reported that zero valence iron, iron oxides, and conductive iron oxides can improve the efficiency of waste degradation and increase methane yield in anaerobic digester systems [83–85]. Hao et al. (2017) used waste iron scraps to enhance degradation efficiency of sludge in a mesophilic fermenter and found that iron scraps stimulated acetogenic and homoacetogenic bacteria, which in turn provided substrates for methanogens [86]. Though our study was limited due to the absence of methanogenic archaea (discussed below), these results support previous findings that the addition of iron stimulates fermentative bacteria that are beneficial to waste degradation efficiency.

The addition of sulfate simulated little change in the overall microbial community (Table 2; Fig 5). Sulfate concentrations measured at the end of the incubation period in the sulfate-treated microcosms is 2000 ± 100 mg/L sulfate, which was equivalent to the amount of sulfate added. This is reflected in the results of the PERMANOVA test, which showed sulfate addition did not influence microbial community composition (Table 2). These results indicate addition of sulfate did not produce changes in the overall microbial community; however, this does not necessarily indicate that sulfur-related metabolism was not active during the incubations. The genus *Desulfotomaculum* was found in low abundance (~0.001%) in several microcosms at the start of the incubation but no other common sulfate reducing bacteria (SRB) were detected later in the incubation period (data not shown), however, some species of the more abundant genera *Acinetobacter* and *Caldalkalibacillus* have been found to be capable of sulfate reduction [87, 88]. *Caldalkalibacillus* was also most abundant at the start of the incubation (Fig 4B), along with the organosulfur oxidizing bacterium *Brevibacillus* [89]. Common household waste such as paper products and packaging materials can provide a source of sulfate, and sulfate was detected in the simulated leachate prior to treatment addition (Table 1), so SRB may have been active early in the experiment and later outcompeted by fermentative bacteria for carbon substrates. Sulfate reducers have been detected in landfill systems, and laboratory incubations have shown competition between methanogens and sulfate reducers [1, 26]. In addition, sulfate has been shown to stimulate AOM when added to landfill cover soils [90]. Therefore, future work should investigate the addition of sulfate at different times during the incubation period, as well as different sources and/or concentrations of sulfate to better understand the influence of sulfate on MSW degradation.

The absence of methanogens in our microcosms warrants potential adjustments to the experimental design such as adding a mechanism for microcosm liquid recycling and neutralization or the addition of a seed of anaerobic bacteria and/or methanogenic archaea [18, 91]. Previous work has found that methane production rates are highly variable in a laboratory setting, and leachate recycling and neutralization is an effective method to stimulate methane production [18, 92]. The leachate recycling and neutralization technique was not used here. The pH at the end of the experimental period of 65 days was 5.5, which would have inhibited the onset of methanogenesis in the absence of acid-tolerant methanogens [6]. This result indicates a potential limitation of this experimental design, as the 100 mL glass bottles were not set up to redistribute liquid collected at the bottom of the microcosms. This should be a consideration for future studies implementing a lab-built waste design.

Our results show that microcosms built with simulated fresh MSW may be an effective tool for observing microbial dynamics to single variables in a heterogenous system over time, with the suggested adjustments to the experimental design to ensure the detection of methanogens. Here, we have tested a small subset of the potential variables that may affect landfill microbiology and waste degradation and found that insoluble iron oxide increased microbial diversity and stimulated fermentative bacteria, while antibiotics shifted the microbial community without influencing microbial diversity. Testing various concentrations and species of sulfur/iron/antibiotics may also be of value as the concentrations of these different waste streams vary in space and time in landfill ecosystems. Future microcosm work would benefit from the incorporation of metagenomic analysis or functional gene testing, as well as produced gas composition analysis to connect changes in the microbial community to metabolic functional changes that would impact waste degradation trajectory.

## 5. Conclusions

Landfill ecosystems are inherently heterogeneous, and while this may present challenges for reproducibility, our results show that microcosms built with simulated fresh MSW can be used to track changes to the landfill microbiome in response to contaminants of concern. Both the antibiotics and iron treatment additions altered the microbial community composition of the microcosms. Some resident microbial taxa within fresh MSW may be replaced with more resistant taxa following a single antibiotic dose. Further work investigating the long-term impact of exposure to antibiotics and various antibiotic dosages on the development of antibiotic-resistance genes and their influence on waste-degrading microorganisms is needed. The influence of iron-bearing metal contamination significantly increased microbial diversity and stimulated fermentative bacteria within the microcosms. Metagenomic or metatranscriptomic sequencing to confirm the metabolic genes present/and or utilized within MSW would be of great value to understanding the dynamics and waste degradation potential of landfill microbial communities.
